# An Exploration of the Value of Elective Health Checks in UK Zoo-Housed Gibbons

**DOI:** 10.3390/ani10122307

**Published:** 2020-12-05

**Authors:** Tawny Kershaw, Emily J. Hall, Phillipa Dobbs, Matyas Liptovszky, Victoria Strong

**Affiliations:** 1 Brackenhurst Campus, School of Animal Rural and Environmental Sciences, Nottingham Trent University, Southwell, Nottinghamshire NG25 0QF, UK; tawny.kershaw2013@my.ntu.ac.uk (T.K.); emily.hall@ntu.ac.uk (E.J.H.); 2 Twycross Zoo East Midlands Zoological Society, Burton Road, Atherstone, Warwickshire CV9 3PX, UK; phillipa.dobbs@twycrosszoo.org (P.D.); liptovszky@gmail.com (M.L.)

**Keywords:** preventative health, routine health examinations, anaesthesia, risk–benefit analysis, *Hylobatidae*, zoological medicine

## Abstract

**Simple Summary:**

The health of zoo-housed animals is of critical importance for their welfare and conservation. Since many wild species often do not show signs of illness, zoo veterinarians routinely carry out health checks in seemingly healthy individuals to identify issues requiring treatment. The benefits of these “elective” health checks must be balanced against the risk of injury or stress, and their financial cost. This study involved the analysis of data gathered from 74 examinations carried out at one UK zoological collection, on 33 gibbons between 2011 and 2018. More than half (*n* = 38) of these health checks resulted in veterinary intervention and/or a management change to the gibbon’s care (referred to as an actionable outcome). Age was found to be an important and reliable determinant of health check outcome, with health checks in elderly gibbons being 13.64 times more likely to have an actionable outcome than those in non-elderly gibbons. X-ray abnormalities, such as osteoarthritis, were also 34 times more likely in this group. Zoo managers and veterinarians can use the methods and findings outlined in this paper to quantify the benefits of elective health checks in their own collections, and to inform evidence-based decision making about the frequency and intensity with which they are implemented.

**Abstract:**

Elective health checks form an important part of the preventative healthcare of many zoo-housed animals. These procedures are not without risk or financial expenditure, meaning careful cost–benefit analysis is required when determining the frequency and intensity with which they are implemented. This study evaluated the value of elective health checks (*n* = 74) carried out on 33 gibbons at a single UK zoological collection from 2011 to 2018. Data were categorised by health check type, animal age, clinical findings and outcome. Univariable binary logistic regression and multivariable modelling were used to identify factors associated with the likelihood of actionable (clinically significant) outcomes. In total, 51.35% of all elective health checks resulted in an actionable outcome. Elderly heath checks had 13.64 times greater odds of an actionable outcome and 34 times greater odds of a significant radiographic finding, when compared to routine (non-elderly) health checks. Our findings suggest that 75% wild longevity is a suitable threshold for identifying elderly captive gibbons and increasing health check frequency. Whilst further work is needed to ascertain whether these findings can be extrapolated to other collections and/or species, this study demonstrates how the analysis of clinical data can aid in the implementation of an effective and evidence-based preventative healthcare plan.

## 1. Introduction

Of the 28,000 endangered species on the planet, few are facing such dramatic population decline as *Hylobatidae*: the family that encompasses all species of gibbon [1]. The maintenance of healthy and self-sustaining captive populations is therefore of critical conservation importance.

The ability to identify and act on health concerns is essential to ensuring the optimal care and welfare of any (wild or domestic) captive animal [2,3]. Whilst informative and important, there are limitations to observational assessment as a health monitoring tool in zoos, not least the heavy reliance on keeper recognition and reporting of issues, and the fact that many zoo animals do not show overt signs of disease [4]. Many zoos have therefore incorporated proactive health checks to monitor the health of their animals into their preventative healthcare plans [5], although the frequency with which these are carried out varies between collections. Zoo animals, including many non-human primates (NHP), can be successfully trained to voluntarily participate in a variety of procedures from cardiac auscultation to radiography [6,7,8,9,10,11], though this is not without limitation and requires heavy investment in terms of staff time. Capture and anaesthesia therefore remain necessary for a complete health examination to be carried out for most zoo-housed NHPs.

Whilst helping to ensure both patient and staff safety [12], capture and anaesthesia are not without risk. They can be associated with acute stress, injury, and loss of social group status [13] and carry a risk of peri-anaesthetic morbidity and mortality. For humans [14] and many domestic animal species, such as dogs, rabbits and cats [15,16,17,18,19,20,21], the risks associated with anaesthesia have been well studied. With the exception of two published studies in gibbons [22] and great apes [23], studies quantifying anaesthetic risk in NHPs are comparatively lacking. As well as carrying an inherent risk, anaesthesia and health checks are financially costly to the zoological institution, and require heavy investment in equipment and facilities, drugs, consumables, and staff time. It is therefore prudent that veterinary practitioners carefully weigh up the pros and cons when deciding upon the frequency and intensity with which such elective health check procedures are carried out.

The benefits of elective health checks in early disease detection have been well documented in dogs and cats [24,25,26,27,28,29]. One published study has also evaluated the effects of elective health checks on animals housed at a UK zoological collection [30]. The authors reported that at least one problem was identified by the health check in almost half (45.7%) of the >1651 cases examined, with subsequent action required in 21.1% of health checks overall. However, these health checks were carried out in amphibians, reptiles, birds and various mammal species. There are no similar studies reporting on the relative value of carrying out elective health checks under anaesthesia specifically in NHPs, making it difficult for veterinary practitioners to draw a clear evidence-based, risk–benefit conclusion on the need for and frequency of these procedures in these species.

This study involved a retrospective survey of elective health check procedures carried out in gibbons housed at a UK zoological collection, which has carried out proactive preventative health check examinations under general anaesthesia as a health monitoring tool since 2011. Under their current preventative healthcare programme, health checks involving a full clinical examination, dental inspection, haematology and biochemistry, rectal swab for culture, whole body radiographs, abdominal +/− cardiac ultrasound, and intradermal tuberculosis testing where appropriate, are carried out at least every two years. The frequency of the health checks is increased to annually and additional radiographs of the joints are taken in elderly animals. The current system used by this zoological collection relies on longevity data (see Table 1) with elderly animals (>75% life expectancy; wild if available, captive if not; based on the zoo’s internal protocol) receiving annual as opposed to biannual health checks (S. Chapman, personal communication).

The aims of this study were to: (1) evaluate the effectiveness and clinical benefit of this programme; (2) test the suitability of the criteria currently used by this collection to identify elderly gibbons and thereby the frequency with which health checks are carried out and; (3) identify those variables which have the greatest effect on actionable health check outcomes.

## 2. Materials and Methods

The study population was defined as any gibbon (*Hylobates agilis,* Agile gibbon; *Hylobates lar,* Lar gibbon; *Hylobates muelleri,* Mueller’s gibbon; *Hylobates pileatus,* Pileated gibbon; *Nomascus leucogenys,* Northern white-cheeked gibbon; and *Symphalangus syndactylus,* Siamang gibbon) that underwent a health check procedure at a single UK zoological collection between 17 March 2011 and 6 November 2018.

Clinical records for each individual and health check were extracted from the Species360 Zoological Information Management System (ZIMS, 2018, zims.Species360.org) and evaluated. Elective health checks were defined as those which were carried out at a predetermined time to monitor the health status of individuals as part of the zoo’s preventative healthcare programme. This included some individuals undergoing health checks at the same time as other planned procedures (e.g., microchip or contraceptive implant placement). Health checks on animals with known long-standing health conditions, such as osteoarthritis, were considered elective provided the animal was clinically well at the time of the examination. Any health checks carried out on a date earlier than planned to investigate concerns about deterioration or possible ill health, or to administer treatment to an animal were excluded. Each health check was recorded as one datapoint.

For each health check, the following information was extracted and recorded: animal identification, species, body condition score (BCS, out of 9), sex, age, reason for health check, any surgical or medical interventions carried out, any diagnostic procedures carried out (radiography, ultrasonography, blood tests, faecal swab culture), the results of these procedures and any action taken as the result of the health check findings. Each health check was categorised according to whether it resulted in an actionable (clinically significant) outcome, defined as any medical intervention (including euthanasia), surgical intervention (including dental) or management change to the individual’s care carried out as a result of the health check findings. Treatments administered alongside preventative healthcare procedures (e.g., analgesia for contraceptive implant placement) or to animals that sustained an injury as part of the health check process, were not classified as actionable outcomes.

Age was assessed as a categorical variable; animals in the first decade of life were split into two groups (0 to < 6 years; 6 to < 10 years) to separate those which were likely to be at pre- and post-sexual stages of maturity [34] and to allow for relatively equal distribution of animals across age categories. Thereafter, animals were grouped into 10-year age intervals and then a group for those aged 40 years or older. The use of quartiles of life expectancy as published elsewhere [30], was not used due to possible variation in longevity between species. Age data were assessed for normality using the Kolmogorov–Smirnov test.

Health checks were categorised by type, based on previously published definitions [30]. Categories were pre-export, first and routine (see Table 2). No post-import health checks were carried out during the study period. Routine health checks were further denoted as routine (non-elderly) and routine (elderly), based on the collection’s classification system as described in Table 1. Hereafter, routine (elderly) health checks are referred to only as elderly. The proportion of each health check type resulting in an actionable outcome was calculated with 95% confidence intervals (95% CI) generated using Epitools (https://epitools.ausvet.com.au/ciproportion. accessed on 6 November 2020).

Diagnostic test results were categorised using a grading system (0–2) developed for this study, with 0 equating to normal results/no abnormalities, 1 pertaining to an abnormal result/finding of no clinical relevance and 2 pertaining to an abnormal result/finding with clinical significance. Categorisation was based on clinician interpretation as reported in the clinical notes by the responsible veterinarian. It was carried out by one individual (T.K.) and checked by a second individual (V.S.) for accuracy and consistency. Table 3 details further definitions and examples for individual categories.

Sample size calculations estimated that cross-sectional analysis would require 65 health check events (including 29 elderly heath check events) to provide a 10.0 odds ratio estimate for an outcome expected to occur in 20% of the routine health check events (5:4 ratio of routine to elderly). Univariable logistic regression analysis was therefore deemed to have an appropriate level of statistical power.

Univariable binary logistic regression modelling was carried out using SPSS 26 (IMB Inc., Armonk, New York, NY, USA) to identify those factors that influenced heath check outcome (actionable outcome as the outcome of interest). Those liberally associated (*p* < 0.2) with a health check outcome were taken forward to multivariable logistic modelling, where more than one factor was identified as being associated with the outcome of interest. As age category was a factor of primary interest, variables that were highly collinear with age category (health check type) were not included in multivariable models with age category but were included in alternative models. Akaike information criterion (AIC) was used to select the final model, and the explanatory ability of the model was evaluated using the area under the receiver operating characteristic (ROC) curve [35] and R^2^ value, alongside consideration of the underpinning biological plausibility of the model specification. Additionally, using the same method, binary logistic regression was used to identify variables associated with a clinically significant radiographic finding, to explore the value of routine radiographic imaging during routine health checks. Statistical significance was set at *p* < 0.05, and odds ratios were determined via logistic regression analysis.

## 3. Results

A total of 74 elective health checks were carried out on 33 gibbons during the study period (Table 3).

### 3.1. Age and Sex Distribution

Forty two percent (*n* = 31) of the health checks were in females and 58% (*n* = 43) in males. Age data were not found to be normally distributed. The median age of the gibbons was 17.83 years (range: 1.33 to 51.69 years). Table 4 shows the distribution of ages by age and species.

### 3.2. Health Check Type, Diagnostic Procedures and Outcome

In total, 38 (51.35%) of all health checks had an actionable outcome. The proportions of health checks resulting in an actionable outcome for each health check type are summarised in Table 5.

In total, 21 health checks resulted in actionable outcomes following a clinically significant finding being identified on diagnostic tests, with an additional 17 actionable outcomes resulting from clinical (including dental) examination findings alone. Details regarding the number and proportion of diagnostic tests that revealed incidental and clinically significant abnormalities are shown in Table 6.

Clinically significant findings identified on blood sample included: band neutrophilia (*n* = 6), azotaemia (*n* = 2), hypoproteinaemia (*n* = 1) and elevated cardiac troponin (*n* = 2). The most frequently identified clinically significant radiographic abnormalities were newly diagnosed or worsened osteoarthritis (*n* = 3) and spondylosis (*n* = 5), or a combination of the two (*n* = 5). Other radiographic abnormalities identified included a tibial fracture, conformational abnormalities, lung infiltrates, cardiomegaly and the presence of an abdominal mass. Echocardiography identified cardiac disease in three animals. Abdominal ultrasound allowed examination of an ovarian cyst in one animal (mass first detected by radiography) and the identification of an intrapelvic mass in another.

Of the 38 health checks that had an actionable outcome, 31 (81.58%) resulted in some form of veterinary intervention with or without concurrent increased monitoring and/or management changes. The remaining health checks resulted in increased keeper and/or veterinary monitoring and/or a change to their management or training regime (Table 7). Of those health checks with an actionable outcome (excluding euthanasia), 14 (41.18%) resulted in a single intervention, 9 (26.47%) were associated with two interventions, 9 (26.47%) resulted in three interventions, one (2.94%) in four and one (2.94%) in five interventions. In addition, five animals required veterinary treatment of small or superficial wounds which, from their appearance, were thought to have been sustained during the catching, restraint or recovery process.

#### 3.2.1. Variables Predicting a Clinically Significant Radiographic Abnormality

**Figure 1:**
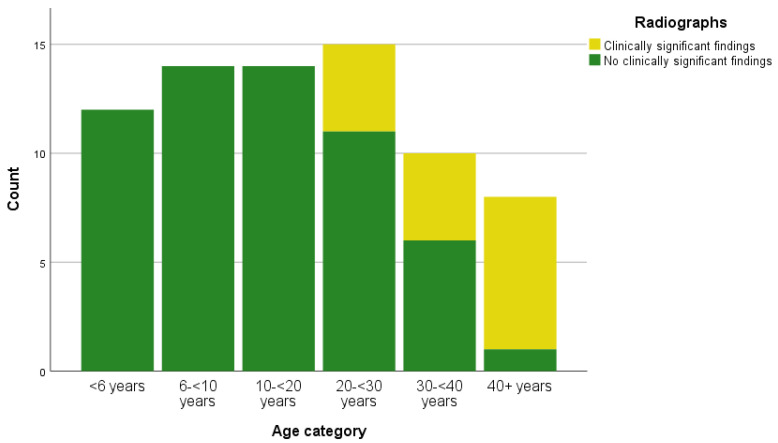
Histogram of radiographic outcomes by age category.

The frequency of significant findings resulting from routine radiographic examination in each of the age categories is shown in Figure 1.

The variables age category (*p* < 0.001), species (*p* = 0.002), BCS (*p* = 0.057) and health check type (*p* < 0.001) were found to be liberally associated with a significant radiographic finding during elective health checks (see Table A1 (Appendix A) for univariable results). At univariable level, animals undergoing routine elderly health checks had 34 times greater odds of significant radiographic findings, when compared to animals undergoing elective routine health checks (see Table A2).

Following model exploration using the AIC, the final model retained one variable: age category, and showed good discrimination (R^2^ = 0.627, area under the ROC curve: 0.920). In the final model, animals aged 20 - < 30 years had 0.05 times the odds of a significant radiographic finding, when compared to animals aged 40 years or older (see Table 8).

#### 3.2.2. Variables Predicting an Actionable Health Check Outcome

Age category (*p* < 0.001) and health check type (*p* < 0.001) were the only variables found to be strongly associated with an actionable health check at univariable level (see Table A1 for all univariable results). Following model exploration using the AIC, the final model retained one variable: age category, and showed good discrimination (R^2^ = 0.438, area under the ROC curve: 0.819). In the final model animals aged 20 - < 30 years had 13.75 times the odds of an actionable health check outcome, all the animals aged 30 - < 40 years had elective health checks that resulted in an actionable outcome, and animals aged 40 years or older had 15 times the odds of an actionable health check outcome when compared to animals aged under 6 years (see Table 9).

## 4. Discussion

Elective health checks are carried out as part of preventative healthcare programmes in zoos housing non-human primates across the globe. There is, however, no clear consensus on the frequency with which they should be implemented. Furthermore, clear, quantifiable evidence of their benefits is largely missing from the veterinary and zoological literature.

This study set out to evaluate the effectiveness and value of elective health checks carried out on six gibbon species housed at a UK zoological collection. Given that more than half (51.35%; *n* = 38/74) of all elective health checks resulted in an actionable outcome, our findings suggest that these procedures are of benefit overall. We present evidence, however, that the relative value of these checks and the diagnostic tests they include, varies according to health check type and, especially, animal age.

### 4.1. Gibbon Age and Health Check Type

We show that increasing age is the most important factor associated with higher likelihood of an actionable outcome (see Table 9). This is not at all surprising given that age is a well-documented risk factor for disease in humans and domestic pets [24,27,36,37]. We therefore support the conclusion also drawn by Barrows et al. [30] that the frequency of health checks an animal receives should increase with age. The question remains, however, as to how zoos should determine at what age this increase in testing should commence.

The current system used by this zoological collection relies on longevity data (see Table 1) with elderly animals (>75% life expectancy; wild if available, captive if not; based on the zoo’s internal protocol) receiving annual as opposed to biannual health checks. Given that most mammals tend to live longer in captivity [38], the use of wild longevity to identify elderly zoo animals has been criticised in the literature [39]. Indeed, using this approach means that four out of the six species included in this study are categorised as elderly from around 20 years of age (range: 19–23 years). The much higher captive life expectancy (38–60 years; Table 1) means that these animals therefore spend a significant proportion of their lives in the elderly category and undergoing annual health checks. These procedures are not without risk of iatrogenic injury, anaesthetic death and/or stress to the animals concerned [13] and incur significant costs for the institution. As part of this study, therefore, we aimed to critically evaluate whether this cut off is in fact, a little premature.

Our findings show, however, that the system currently in place in this zoological collection (elderly: >75% wild longevity) is effective and appropriately identifies animals that benefit from increased health monitoring. As shown in Table 9, gibbons aged 20 ≤ 30 years had 13.75 times the odds of an actionable outcome when compared with gibbons <6 years of age. For comparison, there was no significant difference in the odds of an actionable outcome from health checks in gibbons aged less than 6, 6 ≤ 10 and 10 ≤ 20 years. To further support this, 85.71% of elderly health checks resulted in an actionable outcome (Table 5), and elderly heath checks had 13.64 times the odds of an actional outcome when compared to routine (non-elderly) health checks.

Our findings do, however, highlight one potential flaw in the system used by this collection. Given the lack of data relating to Lar and Mueller’s gibbons’ wild lifespan, these species are not currently classified as elderly until 42 and 45 years of age, respectively. In contrast to what is argued by Vogelnest and Talbot (2019) [39], our findings suggest that using captive life expectancy to identify elderly animals might cause health issues to be missed or detected much later. We therefore propose that a more suitable approach would be to consider any gibbon for which wild longevity data are not available as elderly once it reaches 20 years of age.

Our findings appear to suggest little value in “first” health checks with none of the three carried out resulting in an actionable outcome, although sample size (*n* = 3) was very small. Furthermore, first health checks typically coincide with other important interventions such as the placing of a microchip and determining the animal’s sex and provide veterinarians with valuable baseline clinical information. The decision to carry them out must be made on case-by-case basis. By contrast, a surprisingly high proportion (43%; *n* = 3/7) of pre-export health checks in this study identified an issue which required surgical (dental) or medical intervention. This figure is slightly higher than previously reported in a similar multi-species study [30] albeit from a smaller sample, and highlights the importance of animals receiving full health checks as well as any required infectious disease screening before being transferred to a new collection.

The benefits of carrying out routine (non-export associated) elective health checks in non-elderly adult animals is, however, less clear cut. In our study, issues were identified in less than one third (*n* = 11/36) of cases. For comparison, a previous similar, multi-species study at a different UK zoological collection, reported that at least one problem was identified in 52.2% of all animals, and 58.5% of mammal health checks [30], although not all required subsequent action. The figure from our study is similar to that reported in domestic dogs and cats, with the incidence of disorders such as dental disease and obesity in apparently healthy animals being reported as between 21% and 39% [25,26,27].

The sole focus of this study, the possibility that an elective health check will identify a previously undiagnosed issue or have another clinically significant outcome, is just one factor to be considered when determining the frequency with which such procedures should be carried out. This must be balanced against the potential for the procedure to have a negative impact on the animals’ health and welfare (anaesthetic death, injury, stress) as well as the costs associated with drugs, laboratory fees, facilities, equipment and staff time. For humans [14] and many domestic animal species including dogs, horses, cats and rabbits [15,16,17,18,19,20,21,40], the risks associated with anaesthesia have been well studied. Data relating to anaesthesia-related risks in non-human primates are, however, comparatively lacking. A retrospective survey of >1180 anaesthetic records of zoo-housed great apes reported a peri-anaesthetic mortality risk of 1.35% and identified health status and age as key risk factors for death [23]. A study carried out by Turner et al. (2018) [22] explored peri-anaesthetic mortality among the same captive gibbon population as that studied here. The authors reported one death in 111 procedures (0.9%), although this was in a gibbon undergoing unplanned anaesthesia for a clinical issue. Lambeth et al. (2006) demonstrated differences in physiological measures associated with the stress response in chimpanzees anaesthetised by voluntary versus involuntary injection [10]. Otherwise, there is, however, a paucity of literature quantifying the risk not only of death but also of physiological stress and iatrogenic injury associated with capture, restraint and anaesthesia of non-human primates. Given this lack of evidence, and since resource availability is likely to vary greatly between collections, zoos should be encouraged to monitor and audit the effectiveness of their own processes and protocols, in order to come to an evidence-informed decision, and to ultimately contribute to the development of evidence-based health monitoring protocols.

### 4.2. Health Check Outcomes

Although hereby grouped together, not all actionable outcomes are equal in terms of their impact on animal health and welfare. In instances where a new, progressive and painful condition such as osteoarthritis or spondylosis was diagnosed, the clinical benefit to the animal in starting long term analgesia therapy is clear. The early detection of other progressive conditions such as renal failure also allows for medical and husbandry interventions designed to slow deterioration, to be put in place, thereby maximising both quality and quantity of life. Any improvement to animal health and welfare is also likely to have a positive impact on breeding success [41,42] and, by encouraging more species-specific natural behaviours, might also enhance visitor perception and experience [43].

Post-mortem studies have shown elderly animals to be affected by painful conditions which were not necessarily apparent ante-mortem [44]. The ability of elective health checks to detect these issues and dictate, not only the need for treatment, but in some instances, also humane euthanasia (as in four cases in this study) is therefore key in the prevention of suffering. Determining the appropriate point at which euthanasia should be performed can be very difficult and might sometimes unveil conflicting interests of the zoo directors, keepers, visitors and breeding programme coordinators [45]. Objective interpretation of clinical information gleaned from health checks can, therefore, be very powerful in helping vets to determine the most suitable outcome for the animal’s future wellbeing.

Whilst it was not an objective of this study to evaluate the frequency of the adverse effects of the health checks carried out, it should be mentioned that we did identify five animals (6.8% of all elective health checks) that were administered treatment for minor wounds (superficial lacerations to the hands or feet). From the appearance of these wounds, it was assumed that these were sustained during the catching, restraint and/or recovery process. These findings are a reminder that health checks are not without risk, and that every effort should be made to minimise the likelihood of iatrogenic injury occurring, for example through appropriate facility design and/or training animals for voluntary cooperation with health assessments. In 2016, a new gibbon housing facility was instated at this zoological collection, the design of which facilitates catch-up processes and decreases the need for darting of these animals in the enclosure. Given that hand injection is reportedly associated with improved quality of anaesthetic induction, reduced stress and lessened risk of iatrogenic injury than remote delivery [7,10,22,46,47], it would be interesting to study the impacts of this enclosure change on the frequency of adverse health check outcomes over time. No significant adverse anaesthetic events were reported in our study.

### 4.3. Alternatives to Regular Elective Health Checks

If a collection were to conclude that frequent, elective health checks were of limited clinical value or associated with too high risk and/or cost, they might opt to rely more heavily on gathering clinical information through positive reinforcement training. Indeed, non-human primates can be successfully trained to allow the collection of biological samples such as urine and blood [6,7,8,9,10,11]. In our study, however, the intervention(s) carried out in 17 (45%) of the health checks was based upon clinical examination findings alone. Where this observation was, for example, a gibbon being over/under condition (*n* = 6), conscious weighing and assessment of body condition score might have prevented the need for a general anaesthetic either altogether or may have delayed it until after a diet change had first been trialled. Training for behaviours such as open-mouth presentation and cardiac auscultation would also have allowed for cases of dental disease or suspected cardiac disease to be identified via conscious examination, although subsequent general anaesthesia would have still been necessary for surgical intervention and/or further investigation to be carried out.

If resources for veterinary care are limited, non-elderly animals with no underlying health conditions would be the most suitable group in which to adopt this more conservative and targeted approach to health screening. Collections might opt simply to carry out health checks opportunistically (e.g., to coincide with enclosure moves, contraceptive implant placement) or less frequently than the biannual system hereby discussed. Larger datasets than that presented in this study would be required to prospectively model the impact that reducing the frequency (e.g., to every 3 or 4 years) might have. The effects of any such changes implemented should, therefore, be monitored and audited closely.

### 4.4. Diagnostic Tests

As well as determining the frequency with which health checks are carried out, zoo managers and vets must also determine which diagnostic tests they should incorporate. This decision must take into consideration any potential negative impacts on the animals and the financial costs associated with each test carried out. Given the wealth of evidence that the risk of peri-anaesthetic morbidity and mortality increases with anaesthetic duration [15,40,48,49,50,51], the time taken to carry out each diagnostic procedure is also another very important consideration. The duration of gibbon anaesthetic procedures in this collection range from 15 to 125 min [22] but for preventative health checks in healthy animals, they typically last less than 45 min (P. Dobbs, personal communication). The potential risk of repeating a capture and anaesthetic procedure to perform additional diagnostic tests at a later date must also be considered in any risk/benefit analysis.

Haematology and biochemistry identified a clinically significant issue in 12.5% (*n* = 11) of cases, with additional tests (cardiac biomarkers) providing valuable additional diagnostic information for a further two animals. Especially when carried out under general anaesthesia, venepuncture is associated with minimal risk [52] and animal impact, is quick and easy to carry out and is therefore a valuable component of an elective health check. By comparison, culture and sensitivity findings from rectal swabs resulted in an actionable outcome in only two (<3%) cases, perhaps suggesting this test to be of limited clinical value. However, whilst a rectal swab culture might not on its own, result in an actionable outcome, it might influence therapeutic decision making and therefore be of clinical value. An example of this might be using the results from rectal swab culture and sensitivity to guide antibiotic choice when treating an animal also diagnosed with a band neutrophilia. We therefore conclude that whilst our data do not necessarily support the routine inclusion of rectal swabs in elective health checks, they might still be deemed important and therefore be carried out be at the clinician’s discretion.

An abdominal mass was diagnosed using abdominal ultrasonography in two elderly female gibbons in this study, suggesting that its targeted inclusion in some health checks might be of value. Indeed, it is a non-invasive and low risk procedure that can provide valuable baseline information for the clinician. However, we found no evidence that it identified clinically significant abnormalities in any other (i.e., non-elderly) animals, raising questions about clinical value as a screening tool in elective checks. Veterinarians must therefore carefully consider its routine use and weigh up any potential benefits against the risk of prolonging anaesthetic duration. Although echocardiography identified cardiac disease in three animals in this study, it was carried out in response to clinical suspicion. There is, at present, no scientific evidence to suggest that gibbons are at high risk of cardiovascular disease and so there is insufficient indication that echocardiography should routinely form part of all elective health checks in gibbons.

Our findings suggest radiography is only useful in animals over 20 years of age, the youngest animal with a significant radiographic finding being 21.27 years. Radiography is of most value in animals aged 40 years or over, with animals in this group having 20 times the odds of a significant radiographic finding when compared with those aged 20 ≤ 30 years. Unlike other diagnostic tests such as rectal swab culture, haematology and biochemistry, the routine inclusion of radiography in elective health checks has implications both for staff health and safety (radiation exposure) [53] and increased anaesthetic time. Based on the findings of this study, therefore, veterinarians may consider omitting radiographs from elective health checks of non-elderly animals, to minimise risk to staff and animals alike. As such, they might reserve radiographic examination for cases where there is clinical indication, in elderly animals and/or to obtain one set of diagnostic quality baseline “normal” images for each animal. This conclusion might be particularly relevant for any zoo without portable x-ray or onsite veterinary facilities for whom averting the need to transport the animal to a distant (potentially offsite) facility, will help minimise anaesthetic time, disruption and stress.

### 4.5. Study Limitations

The analysis of retrospective data collected by numerous veterinarians over a period of >7 years undoubtedly has its limitations, not least the potential for inconsistencies in reporting and interpretation of clinical findings. The impact that any subjectivity might have had on the reliability of our results, however, was minimised by using clearly defined criteria and ensuring that the same two individuals (T.K. and V.S.) were responsible for all post-hoc categorisation of data. Although a relatively large dataset for its type, the analysis included multiple species in a single analysis and some categories analysed (for example: first health checks, Mueller’s gibbons) contained only small numbers of individuals, which might limit the generalisability of findings from these groups.

The purpose of this study was to identify potential factors that influenced the elective health check outcome. It aimed to do this through the use of explanatory multivariable logistic regression modelling, rather than attempting predictive statistical modelling. The R2 values for the two models highlight the likely presence of additional factors not considered in this study, and the relatively small numbers of animals in some variables limits the statistical power of these findings. Ideally, the study would be repeated and data from additional years would be included in the study period to generate a larger dataset for predictive modelling purposes. Amongst other things, this would potentially allow species-specific analysis to be performed.

The results of this study do not present analysis of the financial cost of elective health checks or diagnostic testing procedures to zoological collections. The true financial value of elective health checks is likely to vary between collections, not least due to differences in staffing and resource costs, disease prevalence and treatment protocols. Individual collections would, therefore, need to carry out their own cost–benefit analysis. Ultimately, zoos have an ethical responsibility to safeguard the welfare of the animals under their care. Identification of underlying disorders might help to manage or improve the welfare of a single individual animal but may also improve the welfare of a group of animals if the disorder is infectious or husbandry-related. If an animal is part of a breeding programme, early identification of a hereditary disorder and subsequent appropriate reproductive management could also improve the welfare of future generations. Finally, for elective screening of health disorders to truly be beneficial, there is a need for robust, evidence-based reference ranges for not only the species, but also for different life stages as many physiological parameters vary between adult versus geriatric animals [27,29]. One potential benefit of continuing to perform elective health checks on captive populations would be the generation of data to support the creation of such reference ranges, which in turn may improve the clinical value of the health checks.

## 5. Conclusions

Zoo vets carrying out elective health checks as part of preventative healthcare programmes must do so following careful cost–benefit and risk–benefit analysis. This study demonstrates how the evaluation of clinical records can be used to inform decision making and aid in the implementation of an effective and evidence-based preventative healthcare plan in zoos.

Our findings demonstrate that animal age is an important and reliable determinant of health check outcome and conclude that the frequency and intensity of elective health checks should be increased for elderly individuals. Furthermore, we present evidence that using 75% wild longevity as a threshold above which captive gibbons are considered elderly—an approach currently used by this collection—is effective and fit for purpose.

Whilst further work is needed to ascertain whether the findings of this study can be extrapolated to other collections and/or species, the approach used for analysis can be used by other collections as part of in-house auditing of their own processes and protocols.

## Figures and Tables

**Table 1:** Wild and captive life expectancy data used to calculate 75% longevity for elderly health check protocol currently implemented by this collection.

Species	Wild Longevity (Years)	Captive Longevity (Years)	75% Longevity ^1^
Agile gibbon (*Hylobates agilis)*	25	44	19
Lar gibbon *(Hylobates lar)*	*nd*	56	42
Mueller’s gibbon *(Hylobates muelleri)*	*nd*	60	45
Pileated gibbon *(Hylobates pileatus)*	25	38	19
Northern white-cheeked gibbon *(Nomascus leucogenys)*	28	44	21
Siamang gibbon *(Symphalangus syndactylus)*	30	40	23

*nd:* No data available. ^1^ Calculated from wild longevity, or captive data if not available. Sources: [31,32,33].

**Table 2:** Definitions of health check categories.

Category	Definition
Pre-export	Animal undergoes a health examination prior to transfer to another collection.
Post-import	Animal which is new to the collection, undergoes health examination during quarantine period.
First	A young (<1 year) animal, born in the collection, undergoes a health examination. Procedure typically includes, e.g., microchip placement and determination of sex as well as health assessment.
Routine	Health examinations carried out at a predetermined time to monitor the health status of the animal.

**Table 3:** Definitions and examples of categorising system used for grading the presence, absence and/or severity of abnormalities detected by diagnostic tests.

Category	Grade	Description	Example
Blood results	**0**	A completely normal blood panel result with no abnormalities (highs or lows) of any parameters	
	**1**	Blood results with mild alteration in normal parameters but without detriment to the patient or clinical significance	Raised alkaline phosphatase (ALKP) in growing animal
	**2**	Parameters outside of the normal range with clinical significance	Band neutrophilia in patient with suspected active infection
Radiographs	**0**	No radiographic abnormalities detected	
	**1**	Minor radiographic abnormalities detected but with little/no clinical significance	Presence of small stone in the gastrointestinal tract
	**2**	Radiographic abnormalities with clinical significance	Vertebral spondylosis or osteoarthritis
Ultrasound	**0**	No abnormalities detected	
	**1**	Some abnormalities detected but with little/no clinical significance	A small renal cystic lesion
	**2**	Clinically significant abnormalities detected	Uterine mass, cardiac disease
Rectal swab culture	**0**	No abnormalities detected	Bacterial culture typical of normal faecal flora
	**1**	Some abnormalities detected but with little/no clinical significance	Mild or moderate growth of potentially pathogenic bacteria
	**2**	Significant abnormalities detected with clinical significance	Heavy growth of pathogenic bacteria

**Table 4:** Age and sex distribution of gibbon species included in study population.

Gibbon Species	Number of Health Checks			Number of Animals			Median Age (Years)	Age Range (Years)
	m	f	T	m	f	T		
Agile gibbon	8	6	14	4	2	6	21.98	2.27–50.20
Lar gibbon	2	2	4	2	1	3	33.46	3.54–51.69
Mueller’s gibbon	2		2	1		1	50.18	49.67–50.69
Pileated gibbon	10	6	16	4	2	6	16.91	4.02–37.13
N. white-cheeked gibbon	2	8	10	1	3	4	14.14	3.30–40.62
Siamang gibbon	19	9	28	9	4	13	14.56	1.33–34.80

m: male; f: female; T: total; N.: Northern.

**Table 5:** Frequency of health check types and proportion of health checks resulting in an actionable outcome.

Health Check Type	Number of Health Checks	Number of Health Checks with Actionable Outcome	% of Health Checks with Actionable Outcome	95% CI
Routine (non-elderly)	36	11	30.56	18.00–46.86
Elderly	28	24	85.71	68.51–94.30
Pre-export	7	3	42.86	15.82–74.95
First	3	0	0	0.00–56.15

**Table 6:** Frequency of diagnostic tests performed (% of procedures for which tests were carried out), by outcome significance.

Diagnostic Test	Normal Findings (%)	Findings of Limited Clinical Significance (%)	Clinically Significant Findings (%)	Not Performed
Blood sample	30 (41.67)	31 (43.06)	11 (15.28)	2
Radiographs	44 (60.27)	14 (19.17)	15 (20.55)	1
Ultrasound	62 (87.32)	4 (5.63)	5 (7.04)	3
Rectal swab	69 (97.18)	n/a	2 (2.78)	3

**Table 7:** The number and percentage (%) of health checks which resulted in veterinary intervention, increased monitoring and/or management changes, with examples of each.

Action	Description	Number of Health Checks (%)	Examples
**Veterinary**	Short term medication course	10 (13.51)	Antibiotic course due to neutrophilia or results of rectal swab culture
	Start of new long-term medication	5 (6.76)	Commence pain relief medication due to new diagnosis of osteoarthritis (OA)
	Change to long term medication	3 (4.05)	Increase dose of NSAIDs due to worsening OA, cease prior treatment
	Euthanasia	4 (5.41)	Due to deterioration in health condition, multiple age-related issues
	Surgery	12 (16.21)	Dental procedure (extractions, scale and polish)
	Recommendation	4 (5.41)	Specialist involvement (e.g., cardiac scan, complex dental procedure)
**Monitoring**	Increased keeper +/− veterinary monitoring	15 (20.27)	Mobility monitoring, more frequent health checks, regular weighing
**Management**	Training	6 (8.10)	Commence training for regular weighing or conscious examination
	Nutritional	6 (8.10)	Review of dietary provision due to low or high body condition score (BCS)

**Table 8:** Multivariable logistic regression results for variables associated with significant radiographic findings for gibbons undergoing elective health checks between 2011 and 2018.

Independent Variable	Odds Ratio	95% CI	*p*-Value
*Age category*			
<6 years	*		0.998
6 - < 10 years	*		0.998
10 - < 20 years	*		0.998
20 - < 30 years	0.05	0.00–0.57	0.015
30 - < 40 years	0.10	0.01–1.1	0.060
40+ years	Base		

* Indicates a category where 0% of health checks resulted in an actionable outcome.

**Table 9:** Final logistic regression results for variables associated with health checks resulting in actionable outcomes for gibbons undergoing elective health checks between 2011 and 2018.

Variable	Category	Odds Ratio	95% CI	*p*-Value
Age	<6 years	base		
	6 - < 10 years	2000	0.30–13.51	0.477
	10 - < 20 years	2500	0.39–16.05	0.334
	20 - < 30 years	13,750	2.05–92.04	0.007
	30 - < 40 years	~		0.999
	40+ years	15,000	1.65–136.17	0.016

~Indicates a category where 100% of health checks resulted in an actionable outcome.

## Appendix A

**Table A1:** Univariable logistic regression results for variables associated with a significant radiographic finding during elective health checks carried out on gibbons between 2011 and 2018.

Independent Variable	Number of Radiographic Examinations	% of Significant Findings	Odds Ratio	95% CI	*p*-Value
*Gibbon species*					0.002
Agile	14	28.57	Base		
Lar	4	50.00	2.500	0.26–24.38	0.430
Mueller’s	2	100	~		0.999
Pileated	16	0	*		0.998
Siamang	27	11.11	0.310	0.06–1.66	0.172
N. white-cheeked	10	40.00	1.670	0.30–9.27	0.560
*Sex*					0.923
Female	30	20.00	Base		
Male	43	20.93	1.060	0.33–3.37	0.923
*Age_cat*					<0.001
<6 years	12	0	*		0.998
6 - < 10 years	14	0	*		0.998
10 - < 20 years	14	0	*		0.998
20 - < 30 years	15	26.67	0.050	0.00–0.57	0.015
30 - < 40 years	10	40.00	0.100	0.01–1.1	0.060
40+ years	8	87.50	Base		
*Body condition score*					0.057
3	13	38.46	Base		
4	29	20.69	0.420	0.10–1.75	0.232
5	28	14.29	0.270	0.06–1.24	0.092
6	2	0	*		0.999
7	1	0	*		1.000
*Health check type*					<0.001
Elderly	28	50.00	34.000	4.07–283.85	0.001
Export	7	0	*		0.999
First	3	0	*		0.999
Routine	35	2.86	Base		

~Indicates a category where 100% of health checks resulted in an actionable outcome. * Indicates a category where 0% of health checks resulted in an actionable outcome. N.: Northern.

**Table A2:** Univariable logistic regression results for variables potentially associated with actionable outcomes during elective health checks carried out on gibbons between 2011 and 2018.

Independent Variable	Number of Health Checks	% of Actionable Outcomes	Odds Ratio	95% CI	*p*-Value
*Gibbon species*					0.683
Agile	14	50.00	Base		
Lar	4	50.00	1.00	0.11–9.23	1.000
Mueller’s	2	100	~		0.999
Pileated	16	56.25	1.29	0.30–5.43	0.732
Siamang	28	46.43	0.87	0.24–3.13	0.827
N. white-cheeked	10	50.00	1.00	0.20–5.07	1.000
*Sex*					0.610
Female	31	54.84	Base		
Male	43	48.84	0.79	0.31–1.98	0.611
*Age category*					<0.001
<6 years	12	16.67	Base		
6 - < 10 years	14	28.57	2.00	0.30–13.51	0.477
10 - < 20 years	15	33.33	2.50	0.39–16.05	0.334
20 - < 30 years	15	73.33	13.75	2.05–92.04	0.007
30 - < 40 years	10	100	~		0.999
40+ years	8	75.00	15.00	1.65–136.17	0.016
*Body condition score*					0.33
3	13	61.54	Base		
4	29	58.62	0.89	0.23–3.38	0.859
5	29	37.93	0.38	0.10–1.47	0.161
6	2	50.00	0.63	0.03–12.41	0.758
7	1	100	~		1.000
*Health check type*					<0.001
Elderly	28	85.71	13.64	3.81–48.76	<0.001
Export	7	42.86	1.70	0.33–8.93	0.528
First	3	0	*		0.999
Routine	36	30.56	Base		

~Indicates a category where 100% of health checks resulted in an actionable outcome. * Indicates a category where 0% of health checks resulted in an actionable outcome. N.: Northern.
